# Effects of Antimicrobial Photodynamic Therapy and Local Administration of Minocycline on Clinical, Microbiological, and Inflammatory Markers of Periodontal Pockets: A Pilot Study

**DOI:** 10.1155/2018/1748584

**Published:** 2018-04-05

**Authors:** Takahiro Hokari, Toshiya Morozumi, Yasutaka Komatsu, Taro Shimizu, Toshiaki Yoshino, Maki Tanaka, Yoshie Tanaka, Kaname Nohno, Takehiko Kubota, Hiromasa Yoshie

**Affiliations:** ^1^Division of Periodontology, Department of Oral Biological Science, Niigata University Graduate School of Medical and Dental Sciences, Niigata, Japan; ^2^Oral Implant Clinic, Niigata University Medical & Dental Hospital, Niigata, Japan; ^3^Seikeikai Hospital, Seikeikai Group, Yokohama, Japan; ^4^Division of Preventive Dentistry, Department of Oral Health Science, Niigata University Graduate School of Medical and Dental Sciences, Niigata, Japan

## Abstract

**Objective:**

We evaluated the efficacies of antimicrobial photodynamic therapy (aPDT) and minocycline ointment (MO) on clinical and bacteriological markers and the local host inflammatory response.

**Materials and Methods:**

A total of 30 patients with chronic periodontitis were randomly assigned to two groups. Selected periodontal pockets (probing depth 5–7 mm with bleeding on probing) were treated with aPDT or MO. Measurements of clinical parameters and the collection of gingival crevicular fluid (GCF) and subgingival plaque were performed at baseline, and at 1 and 4 weeks after treatment. Quantification of periodontopathic bacteria in the sulcus and a multiplex bead immunoassay of ten inflammatory cytokines in the GCF were performed.

**Results:**

Local MO administration exhibited a significant decrease in scores for clinical parameters (*P* < 0.01) and a significant reduction in bacterial counts (*P* < 0.01) and interleukin-1*β* and interferon-γ levels at 1 and 4 weeks after treatment (*P* < 0.01). No significant changes were observed in the aPDT group, except in clinical parameters.

**Conclusions:**

Although our study had some limitations, we found that while local administration of MO may slightly help to improve clinical, microbiological, and crevicular cytokine levels in periodontal pockets, aPDT did not show any effects. This trial is registered with the UMIN Clinical Trials Registry UMIN000013376.

## 1. Introduction

Chronic periodontitis is an inflammatory and multifactorial disease caused by periodontopathic bacteria and the host immune response [1]. Scaling and root planing (SRP) is an essential procedure for the treatment of periodontitis and is frequently performed during various phases of periodontal treatment to remove its etiologic agents that cause inflammation, which include dental plaque, bacterial products, and calculus. However, the efficacy of SRP can be limited in cases with less access to deep pockets, root furcations, and concavities [2]. Thus, adjunctive periodontal treatments to SRP have been proposed to eradicate or reduce the numbers of pathogenic bacteria in those areas [3].

Several clinical and bacteriological studies have indicated the possibility of various anti-infectious therapies as adjunctive treatments, including disinfectants and antibiotics [3]. We have also previously reported the antimicrobial effects of essential oil-containing antiseptics [4, 5] and minocycline ointment (MO) as a local drug-delivery system [6, 7], as well as the oral administration of azithromycin [4]. The effectiveness of systemic antimicrobial administration in the treatment of periodontal disease has been demonstrated [3]. However, side effects resulting from overdose or the systemic appearance of drug-resistant bacteria have also been reported [3, 8]. Similarly, laser therapy also might cause irreversible thermal damage to the surrounding periodontal tissue if used at a high power [9].

Antimicrobial photodynamic therapy (aPDT), which involves low-intensity diode laser irradiation along with photosensitizers, is a new method of antimicrobial treatment. Activation of the photosensitizer by irradiation at a suitable wavelength results in the release of singlet oxygen, which interacts with and is toxic to the cells or microorganisms [10]. Minocycline is a semisynthetic derivative of tetracycline, which exhibits a broad antibacterial spectrum [11]. MO exhibits various favorable features, such as marked substantivity, slow-release, and superior lipophilicity [11]. Furthermore, in addition to its antibacterial activity, MO exhibits a therapeutic effect in periodontitis by directly inhibiting collagenase activity [12]. Thus, MO is a preferred antibiotic for periodontal disease control, especially for local therapy.

Biofilm-associated infections are generally difficult to treat with antibiotics (as in anti-infectious periodontal therapy) because the protective characteristics conferred by the dental biofilm structure make it impossible to achieve a correct disorganization and bacterial cell lysis without using a mechanical procedure [3]. Therefore, the general consensus holds that if antimicrobial therapy is considered, it should be preceded by thorough mechanical debridement to disrupt the structured biofilm [13]. Consequently, there are few reports of local application of an antimicrobial agent or light irradiance to the floating bacteria and biofilm surface layer in gingival sulcus in patients who have not yet received SRP. However, using these procedures before mechanical debridement has the potential to be beneficial in some patients. For example, it may be possible to reduce the incidence of bacteremia and obtain a beneficial clinical outcome if these applications are performed before and after SRP in high-risk patients, such as weakened immune system or infective endocarditis. Thus, we first investigated the effects of local antimicrobial therapy alone to understand its local microbiological and immunological effects.

Overall, this present study aimed to evaluate the effects of aPDT and MO on both clinical and bacteriological markers and on local primary inflammatory cytokine levels in GCF from periodontal pockets in patients with chronic periodontitis. Considering all of these factors, one might hypothesize that both aPDT and MO might partially contribute to improvements in subgingival bacterial and local immunological markers.

## 2. Materials and Methods

### 2.1. Subjects

This study was a pilot and clinical intervention study with a 4-week follow-up period. A total of 30 patients diagnosed with generalized moderate-to-severe chronic periodontitis, according to criteria of the Guidelines of the American Academy of Periodontology (moderate: 3-4 mm clinical attachment loss, severe: ≥5 mm loss, generalized: >30% of sites affected) [14], were recruited from two facilities (Niigata University Medical and Dental Hospital and Seikeikai Hospital) in Japan, between March 2014 and October 2015. This study received approval from the Ethics Committee of Niigata University Medical and Dental Hospital (NH25-010 and NH25-010N). Written consent was obtained from all participants. All individuals were greater than 30 years and had no risk factors for periodontitis, such as diabetes and smoking, and possessed at least 20 teeth. Subjects with the following conditions were excluded: pregnancy or breast feeding; acatalasia; glucose-6-phosphate dehydrogenase deficiency; photosensitivity disorders; allergy to tetracycline or methylene blue; and use of systemic photosensitizing agents, antibiotics, or anti-inflammatory drugs within 3 months prior to enrollment. Individuals who had received periodontal therapy within the previous 6 months were also excluded. We used GCF inflammatory mediators as a primary endpoint, whereas other parameters, including clinical parameters and bacterial markers, were secondary endpoints in the study. The power calculation test was performed, setting an effect size = 0.80, *α* = 0.05, and a power at 80% [15, 16]. The sample size calculation showed a requirement for 12 subjects per group. Accordingly, we recruited 15 subjects per group.

### 2.2. Clinical Protocol

**Figure 1:**
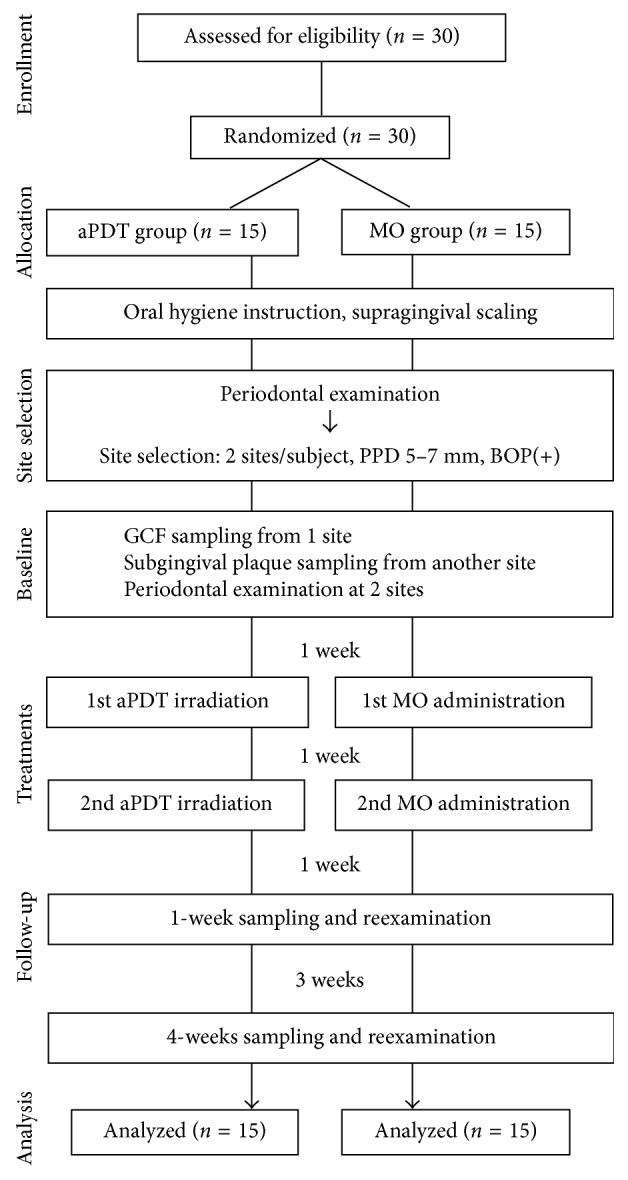
Flowchart of the study from enrollment to completion of the trial.

To evaluate the efficacies of the two therapeutic procedures for periodontal pockets, the 30 enrolled subjects from two facilities were randomly assigned to two groups (aPDT group and MO group; *n*=15, each) on the basis of the treatment protocol and using random tables prepared by one of the authors (TK). Each subject was assigned a code number, which was then used to identify the subject throughout the study. Experimental procedures and data collections were performed in the two facilities between May 2014 and December 2015. A flowchart of the clinical procedure is shown in Figure 1. In brief, for over a month before commencement of the study, each subject received a full-mouth supragingival scaling with an ultrasonic device in a single visit. Subsequently, standard oral hygiene instructions were performed using a toothbrush, interdental brush, and dental floss, according to movies for the fundamental practice of periodontology produced by the Japanese Society of Periodontology, over the course of several visits. All subjects eventually achieved effective individual plaque control and, specifically, a plaque control record <20% within 4 weeks. After the subjects underwent a periodontal examination, two periodontal pockets (probing depth (PD) 5–7 mm, with bleeding on probing (BOP)) were selected for evaluation, such that the pockets were in different, single-rooted teeth and that each tooth was in a different quadrant. For baseline parameters, GCF or subgingival plaque samples were taken from each pocket. Subsequently, the selected pockets—specifically, each selected pocket and its diagonal sulcus—were treated by either aPDT or MO. This treatment was repeated one week later. Subsequent sample collection and periodontal examinations at the two selected sites were performed 1 and 4 weeks after treatment. If any general or oral health problems were reported by subjects or research group members during the study period, the program was aborted. Treatments were performed by one of the two periodontists (TM and TY), who underwent sufficient training to minimize technical differences as much as possible. Calibration of two examiners who were periodontists (YK or MT) were carried out using two different types of periodontal disease models (P15FE-500HPRO-S2A1-GSF, P15FE-500HPRO-S2A1-GSD; NISSIN, Kyoto, Japan) before the start of the study. Full-mouth PD and recessions were measured twice, and intraexaminer repeatability for clinical attachment level (CAL) was assessed. The examiner was judged to have made reproducible measurements after reaching a percentage of agreement within ±1 mm between repeated measurements of at least 95% of measurements. All examiners were blinded to the therapy method used in the individual patient. The two therapeutic procedures are described below.

### 2.3. Antimicrobial Photodynamic Therapy

The periodontal pocket was filled with a 0.01% methylene blue photosensitizer using a blunt needle in a coronal direction starting in the most apical portion. After one minute, the pocket was irradiated for 60 s using a 670 nm wavelength laser (Periowave™ therapy, Ondine Bioharma Corporation, Canada) with an energy dose of 21 J/cm^2^ and power output of 140 mW, in accordance with the manufacturer's instructions [17].

### 2.4. Treatment with Minocycline Ointment

Two percent minocycline gel (PERIOCLINE, Sunstar, Osaka, Japan) was gently inserted into the base of the periodontal pocket and then slowly pulled out in a zig-zag motion while continuing the injection, as described previously [18].

### 2.5. Clinical Assessment

Five clinical parameters were recorded based on periodontal examination: BOP, PD, and clinical attachment level (CAL) at six sites per tooth; plaque index (PlI) and gingival index (GI) at four sites per tooth. The rate of bone resorption was calculated on the basis of the alveolar bone-defect depth measured using dental X-ray radiographs.

### 2.6. Sample Collection

After removing the supragingival plaque on the targeted teeth, GCF collection was performed at one of the sites by consecutively inserting four sterile Periopaper strips (Harco Electronics, Winnipeg, MB, Canada) into the orifice of the gingival crevice until mild resistance was felt; it was then left in place for 30 s per strip. At the second site, two sterile #40 paper points (Zipperer Absorbent Paper Points, VDW GmbH, Munich, Germany) were inserted consecutively into the periodontal pocket for 10 s per point to collect subgingival plaque samples. All strips that absorbed GCF were stirred in 200 *μ*L of phosphate buffer supplemented with 0.5% bovine serum albumin for 15 min at room temperature, which was then centrifuged at 12,000 ×g for 10 min after removing the strips. Supernatants were collected and immediately sent to a medical laboratory (Filgen Inc., Nagoya, Japan) for multiplex array analysis [19]. Meanwhile, subgingival plaque samples were sent to BML Corporation (Tokyo, Japan) for bacterial analysis [5].

### 2.7. Analysis of Inflammatory Mediators

GCF levels of ten inflammatory mediators were assayed using the multiplex assay technique (ProcartaPlex multiplex immunoassays human Th1/Th2 cytokine panel; Affymetrix eBioscience, Santa Clara, CA, USA) according to the manufacturers' instruction manual. The assay was read using a Bio-Plex 200 System (Bio-Rad, Hercules, CA, USA) with the Bio-Plex Manager software v6.0 (Bio-Rad) [20]. To demonstrate a high level of correlation between measurements, duplicate measurements were performed with a subset of samples, for which the intraclass correlation coefficients varied from 0.95 to 1.0 (*P* < 0.001). The following cytokines were measured: interleukin- (IL-) 1*β*, IL-2, IL-4, IL-5, IL-6, IL-12p70, IL-13, interferon (IFN)-γ, tumor necrosis factor (TNF)-*α*, and granulocyte-macrophage colony-stimulating factor (GM-CSF).

### 2.8. Quantification of Periodontal Bacteria from Subgingival Plaques

Quantitative analysis of total and periodontopathic bacterial counts, including *Porphyromonas gingivalis* and *Tannerella forsythia*, was performed using a modified Invader PLUS assay, as described previously [21, 22]. The proportions of the two pathogens compared to total bacterial counts were calculated [23]; the ratio (%) of each species was used for various comparisons as well as for bacterial counts (log_10_).

### 2.9. Statistical Analysis

Data were subjected to descriptive analysis, and the results are presented as mean ± standard deviation. All intergroup comparisons were performed using the Mann–Whitney *U* test, except for gender distribution, which was evaluated using Fisher's exact test. A *P* value < 0.05 was considered to be statistically significant. Intragroup comparisons of clinical, bacterial, and GCF markers at the three time points were performed using the Wilcoxon signed-rank test with the Bonferroni correction, for which the accepted significance threshold was *P* < 0.017. All analyses were performed using IBM SPSS Statistics version 19 (IBM Japan, Tokyo, Japan).

## 3. Results

All participants successfully completed the study protocol, and postoperative healing was uneventful in all cases. None of the subjects reported any general or oral health problems during the study period.  Table 1 shows subject demographic and periodontal data from full-mouth locations at baseline. No significant differences were observed between the two groups for all characteristics.

Table 2 shows intra- and intergroup comparisons of the clinical parameters and subgingival bacterial levels at sites treated with aPDT or MO. Compared with baseline values, the mean scores for PD (mm), CAL (mm), and BOP (% positive) had significantly decreased at 4 weeks after aPDT (*P* < 0.017); the corresponding scores in the MO group had also significantly decreased at 1 and 4 weeks after treatment (*P* < 0.017). Further, the MO group exhibited a significantly lower PD score than the aPDT group at 1 week after treatment (*P* < 0.05). In the aPDT group, no significant difference was apparent in the bacterial count or ratio (bacterial count of each species/total bacterial count) of any of the bacterial species among the various time points. In contrast, the bacterial counts and ratios of both *P. gingivalis* (*P* < 0.01) and *T. forsythia* (*P* < 0.01) had significantly decreased by one week after the application of MO; a significant reduction in *P. gingivalis* counts could still be observed 4 weeks after MO treatment (*P* < 0.01). At 1 week after treatment, the bacterial counts and ratios of both *P. gingivalis* (*P* < 0.001) and *T. forsythia* (*P* < 0.01) in the MO group were significantly lower than those in the aPDT group.

**Figure 2:**
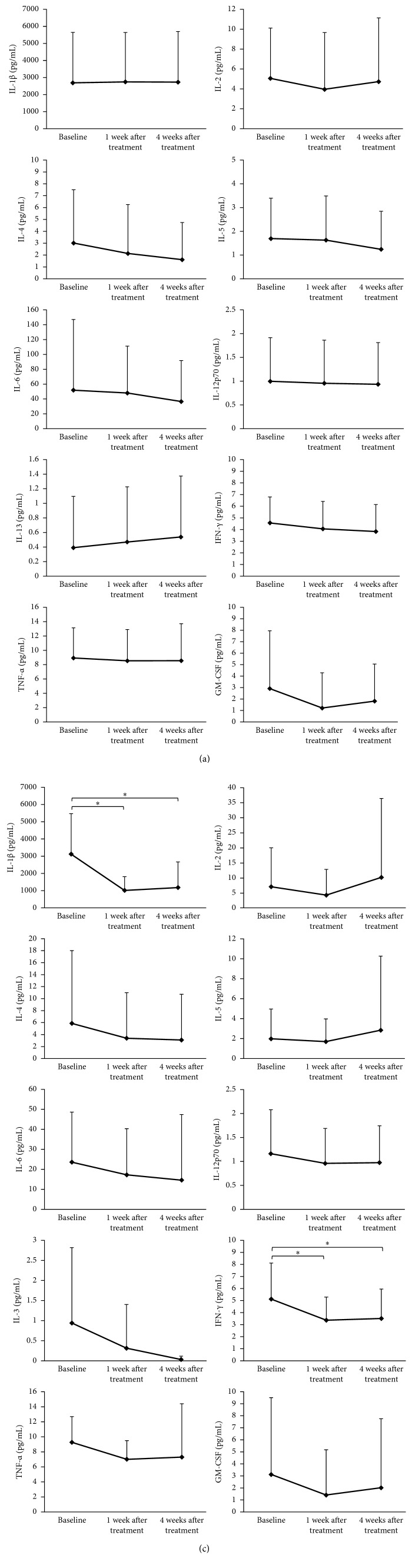
Changes in IL-1β, IL-2, IL-4, IL-5, IL-6, IL-12p70, IL-13, IFN-γ, TNF-α, and GM-CSF levels in gingival crevicular fluid in the aPDT (a) and MO (b) groups. Results are shown as mean ± standard deviation. Data were adjusted for multiple comparisons. ^∗^Significant changes at *P* < 0.017. IL, interleukin; IFN, interferon; TNF, tumor necrosis factor; GM-CSF, granulocyte-macrophage colony-stimulating factor; aPDT, antimicrobial photodynamic therapy; MO, minocycline ointment.

Figure 2(a) presents the levels of GCF inflammatory mediators at baseline and 1 and 4 weeks after aPDT. The levels of all markers, except those of IL-1*β* and IL-13, decreased gradually from baseline, although the decrease was not statistically significant.  Figure 2(b) shows the levels of GCF inflammatory mediators at baseline and 1 and 4 weeks after local administration of MO. Relative to the baseline values, there was a marked decrease in the levels of IL-1*β* (*P*=0.0022) and IFN-γ (*P*=0.0032) a week after treatment. The levels of IL-1*β* (*P*=0.0076) and IFN-γ (*P*=0.0076) had significantly decreased relative to the baseline at 4 weeks after treatment. There were no significant changes in the levels of IL-2, IL-4, IL-5, IL-6, IL-12p70, IL-13, TNF-*α*, and GM-CSF among the three time points. No significant intergroup difference was observed in the level of any of the cytokines.

## 4. Discussion

Generally, the local application of an antimicrobial agent or light irradiance to a periodontal pocket before mechanical debridement in periodontal treatment is not recommended because it is difficult to obtain a sufficient outcome. However, it may be possible to reduce the incidence of bacteremia and obtain a beneficial clinical outcome if these applications can be performed before and after SRP in high-risk patients, such as individuals with a weakened immune system or infective endocarditis. Consequently, in the study, we first investigated the effects of these applications alone as a pilot study to understand their local microbiological and immunological effects because these data had not been previously reported. We demonstrated that MO slightly provides helpful clinical and antibacterial outcomes as well as positive modulation of local cytokine levels. In contrast, aPDT did not yield any effects, at least within the limitations of this study.

We found significant differences in the levels of several GCF cytokines after the local application of MO. Interleukin-1*β* and IFN-γ were the only cytokines that had decreased in concentration, relative to the baseline, one week after treatment and remained at significantly lower levels until the end of the observation. Interleukin-1*β* is known as a representative proinflammatory cytokine that can initiate and regulate the inflammatory response and mediate periodontal tissue destruction; its production and cellular response is partly affected by IFN-γ [24]. To the best of our knowledge, no study to date has reported the effects of MO alone on GCF cytokine levels. Therefore, the present study is the first report to bring changes in GCF cytokine levels in periodontal pockets resulting from MO administration before mechanical therapy. It was interesting to observe a similar tendency of a reduction after therapy in IL-1*β*, IFN-γ, TNF-*α*, and GM-CSF levels, as well as IL-4 and IL-6. It was somewhat surprising that the levels of IL-1*β* were markedly higher than those of other cytokines. Direct comparison of our present results with those of other studies might be difficult because of differences in the evaluation of techniques for selected mediators in GCF between the present and previous studies [25]. A recent study of 14 different immunoassays reported important interassay disparities, as well as marked variations among laboratories, suggesting that the results obtained using a specific assay cannot be compared with those obtained with those of another type of assay [26].

Although MO administration promoted significant changes in bacterial and GCF markers, those of inflammatory markers exhibited minimal changes in the aPDT group. This difference might be attributable to the presence of subgingival calculus. Patients not yet subjected to subgingival scaling and SRP were the subjects of this study. Before commencement of the study, we only removed supragingival plaque and tartar because these bacterial deposits interfere with the insertion of an applicator tip for MO administration, as well as application of the photosensitizer and light illumination into the periodontal pockets. Nevertheless, it remained possible that, during insertion of the laser probe into the base of the periodontal pocket, the tip was obstructed by subgingival calculus and, consequently, laser irradiation failed to adequately produce oxygen radicals. However, it appears that, owing to their physical properties, both MO and the photosensitizer could reach the deeper part of the periodontal pocket. Recently, Kolbe et al. [27] reported monotherapy by aPDT to be advantageous in terms of modulation of cytokines; however, in contrast to the present study, the previous study targeted periodontal pockets that were already treated by SRP. Indeed, de Oliveira et al. [28] also reported the effects of aPDT on crevicular inflammatory mediators in patients with aggressive periodontitis. Aggressive periodontitis exhibits features such as phagocyte abnormalities and a self-limiting disease pattern and is, therefore, different from chronic periodontitis, which was the disease target in the present study [29]. Regarding methodology, it appears to be challenging to achieve an effect with aPDT before subgingival mechanical treatment for periodontal pockets.

A week after MO administration in the present study, subgingival bacterial counts of *P. gingivalis* and *T. forsythia* had reduced by approximately one-hundred-fold and tenfold from baseline levels, respectively. It is interesting that, despite comparable bacterial counts at baseline, there was a substantial difference in the reduction between these two species. Studies involving the local application of MO in patients undergoing supportive periodontal therapy [30] or combination therapy with MO and SRP [31] have also reported a similar tendency. Furthermore, in the present study, only the *P. gingivalis* population continued to remain significantly low at 4 weeks after treatment. Therefore, we believe that *P. gingivalis* might be more susceptible to minocycline treatment than *T. forsythia*. Curiously, our results showed no significant change in the total bacterial count in the MO group. Okuda et al. [32] reported that local delivery of MO causes a reduction in the proportions of periodontal pathogens, such as spirochetes, motile rods, dark-pigmented *Bacteroides* spp., and *Prevotella intermedia*. Consequently, this causes an increase in the proportions of cocci and *Streptococcus* spp., which are considered to be favorable for periodontal health.

In the MO group, we observed significant reductions in PD, CAL, and BOP at the treated sites; notably, the BOP scores had decreased drastically. This might have been a consequence of an increase in tissue resistance to periodontal probing force resulting from a post-treatment improvement in bacterial flora and the subsequent reduction in inflammation in the periodontal pockets [33]. It is somewhat strange that the aPDT group also exhibited reduction in clinical parameter scores. Considering the lack of significant changes in bacterial markers in this group, it is difficult to determine the most probable reason for the reduction in clinical parameters, although one of the possible causes could be the Hawthorne effect.

We are aware of certain limitations of this study. First, antimicrobial therapy should initially be performed as an adjunct to mechanical debridement. Thus, if the test group combined with SRP was compared to another group involving SRP with substances as placebos as a control group, it would be a practical evaluation. Second, the most effective phase of antimicrobial treatment as monotherapy is supportive periodontal therapy (SPT), as specific periodontal sites might not respond to conventional therapies. If we used this study design and evaluation/administration in the SPT, it might show the greatest benefit. Third, the paper point method used to collect subgingival plaque samples in this study is targeted to the floating bacteria and biofilm surface layer in the periodontal pockets. It would be recommended to collect subgingival biofilm samples with mini five curettes and increase the number of sites/samples from different quadrants, to obtain a representation map of the periodontal ecology. Fourth, the primary endpoint should be a clinical parameter because this pilot study involved a clinical trial. Thus, it would require approximately 6 months of follow-up data from a future large-sized clinical trial based on the outcomes of this present study.

## 5. Conclusion

In conclusion, we have demonstrated that local MO administration may slightly help to improve clinical, microbiological, and crevicular cytokine levels in periodontal pockets. Furthermore, no effect was observed after treatment with aPDT within the limits of this study.

## Figures and Tables

**Table 1:** Subject demographics and periodontal data from full-mouth locations at baseline.

	aPDT group (*n*=15)	MO group (*n*=15)
Gender (male/female)	7/8	6/9
Age (years)	61.4 ± 10.2	66.7 ± 9.5
Number of teeth (*n*)	23.3 ± 3.7	24.5 ± 3.6
Bone resorption (%)	24.3 ± 7.0	28.3 ± 9.6
PlI	0.4 ± 0.7	0.5 ± 0.6
GI	0.9 ± 0.7	1.0 ± 0.6
PD (mm)	3.0 ± 0.9	2.9 ± 0.8
CAL (mm)	3.8 ± 1.1	3.7 ± 1.2
BOP (% positive)	27.1 ± 16.5	28.6 ± 15.7

Values are represented as mean ± standard deviation. Gender: Fisher's exact test (*P* < 0.05). Other parameters: Mann–Whitney *U* test (*P* < 0.05).

**Table 2:** Changes in clinical parameters and bacterial levels after aPDT or local minocycline administration.

	(a) Baseline		(b) 1 week after treatment		(c) 4 weeks after treatment		*P* value						
							Between (a) and (b)		Between (a) and (c)		Between aPDT and MO groups		
	aPDT group	MO group	aPDT group	MO group	aPDT group	MO group	aPDT group	MO group	aPDT group	MO group	(a)	(b)	(c)
PD-treated sites (mm)	5.8 ± 1.0	5.4 ± 1.1	5.4 ± 1.3	4.7 ± 1.3	4.8 ± 1.4	4.3 ± 1.4	0.0569	0.0024^†^	0.0003^†^	0.0001^†^	0.1417	0.045^∗^	0.1581
CAL-treated sites (mm)	6.8 ± 1.6	6.1 ± 1.8	6.2 ± 1.8	5.4 ± 1.6	5.8 ± 1.8	5.1 ± 1.7	0.0254	0.004^†^	0.0005^†^	0.0022^†^	0.1809	0.1275	0.2443
BOP-treated sites (% positive)	76.7 ± 43.0	80.0 ± 40.7	66.7 ± 47.9	43.3 ± 50.4	40.0 ± 49.8	46.7 ± 50.7	0.4054	0.0023^†^	0.0116^†^	0.0039^†^	0.756	0.0717	0.6054
Total bacteria (log_10_)	4.72 ± 1.05	4.53 ± 0.87	4.68 ± 0.76	4.16 ± 0.53	4.57 ± 1.11	4.17 ± 0.67	0.8015	0.1228	0.4771	0.2101	0.3826	0.0531	0.0805
*P. gingivalis* (log_10_)	2.63 ± 1.62	2.68 ± 1.44	2.42 ± 1.68	0.61 ± 0.41	2.28 ± 1.79	1.12 ± 1.31	0.8753	0.0033^†^	0.3574	0.0053^†^	0.9331	0.0006^∗^	0.0548
*P. gingivalis* ratio (%)	2.70 ± 3.47	5.80 ± 12.11	3.59 ± 4.39	0.01 ± 0.02	3.20 ± 4.48	1.20 ± 3.49	0.4328	0.0022^†^	0.7213	0.0414	0.6153	0.0006^∗^	0.0518
*T. forsythia* (log_10_)	3.01 ± 1.50	2.89 ± 1.31	2.89 ± 1.54	1.45 ± 0.87	2.87 ± 1.51	2.03 ± 1.23	0.7986	0.0032^†^	0.5627	0.064	0.5883	0.0088^∗^	0.0915
*T. forsythia* ratio (%)	4.08 ± 3.58	4.27 ± 4.60	4.75 ± 4.62	0.77 ± 1.03	4.51 ± 4.30	2.62 ± 4.70	0.7537	0.0019^†^	0.7532	0.2719	0.9834	0.0083^∗^	0.1694

Data are presented as mean ± standard deviation. Ratio was defined as (individual bacteria count)/(total bacterial count). Intergroup comparison between groups: Mann–Whitney *U* test (^∗^*P* < 0.05). Intragroup comparisons: Wilcoxon signed-rank test with the Bonferroni correction for multiple comparisons (^†^*P* < 0.017).
